# Differential impact of mass and targeted praziquantel delivery on schistosomiasis control in school-aged children: A systematic review and meta-analysis

**DOI:** 10.1371/journal.pntd.0007808

**Published:** 2019-10-11

**Authors:** Danielle M. Cribb, Naomi E. Clarke, Suhail A. R. Doi, Susana Vaz Nery

**Affiliations:** 1 Research School of Population Health, Australian National University, Canberra, Australia; 2 Kirby Institute, University of New South Wales, Sydney, Australia; 3 Department of Population Medicine, College of Medicine, Qatar University, Doha, Qatar; Ministère de la Santé Publique et de la Lutte contre les Endémies, NIGER

## Abstract

**Background:**

Schistosomiasis is a widespread public health concern in the poorest regions of the world. The principal control strategy is regular praziquantel administration to school-aged children in endemic areas. With calls for the elimination of schistosomiasis as a public health problem, expanding praziquantel delivery to all community members has been advocated. This systematic review and meta-analysis compares the impact of community-wide and child-targeted praziquantel distribution on schistosomiasis prevalence and intensity in school-aged children.

**Methodology/Principal findings:**

We searched MEDLINE, Embase and Web of Science to identify papers that reported schistosome prevalence before and after praziquantel administration, either to children only or to all community members. Extracted data included *Schistosoma* species, drug administration strategy, number of treatment rounds, follow-up interval and prevalence and intensity before and after treatment. We used inverse variance weighted generalised linear models to examine the impact of mass versus targeted drug administration on prevalence reduction, and weighted boxplots to examine the impact on infection intensity reduction. This study is registered with PROSPERO, number CRD42018095377.

In total, 34 articles were eligible for systematic review and 28 for meta-analysis. *Schistosoma mansoni* was reported in 20 studies; *Schistosoma haematobium* in 19 studies, and *Schistosoma japonicum* in two studies. Results of generalised linear models showed no detectable difference between mass and targeted treatment strategies on prevalence reduction in school-aged children for *S*. *mansoni* (odds ratio 0.47, 95%CI 0.13–1.68, p = 0.227) and *S*. *haematobium* (0.41, 95%CI 0.06–3.03, p = 0.358). Box plots also showed no apparent differences in intensity reduction between the two treatment strategies.

**Conclusions/Significance:**

The results of this meta-analysis do not support the hypothesis that community-wide treatment is more effective than targeted treatment at reducing schistosomiasis infections in children. This may be due to the relatively small number of included studies, insufficient treatment coverage, persistent infection hotspots and unmeasured confounders. Further field-based studies comparing mass and targeted treatment are required.

## Author summary

Schistosomiasis is a neglected tropical disease, caused by parasitic worms, that affects more than 143 million people worldwide. Chronic infections can lead to significant morbidity including kidney damage, anaemia, malnutrition, infertility and growth impairment. School-aged children between six and 15 years are often targeted for regular treatment with praziquantel in large-scale drug delivery programs, because they suffer a disproportionate burden of morbidity. On the other hand, a mass drug delivery strategy that treats all members of the community has been suggested in a move towards elimination of schistosomiasis as a public health problem. In this systematic review, we assess the impact of community-wide versus children-only praziquantel distribution in reducing schistosomiasis infections in school-aged children. We did not detect a difference between mass and targeted treatment strategies, possibly due to factors including insufficient treatment coverage and persistent sources of reinfection. Addressing these factors may assist in optimising control programs.

## Introduction

Schistosomiasis is a water-borne neglected tropical disease (NTD) that infects an estimated 143 million people worldwide [1]. Its global disease burden is estimated at 2.5 million disability-adjusted life years, and 220 million people across 52 countries live in areas endemic for schistosomiasis [1, 2]. The disease is caused by parasitic trematodes of the *Schistosoma* genus, hosted in freshwater *Bulinus* snails, and manifests in intestinal (*Schistosoma mansoni*, *Schistosoma japonicum*, *Schistosoma mekongi*, *Schistosoma guineensis* and *Schistosoma intercalatum*) and urogenital (*Schistosoma haematobium*) forms [3]. Transmission occurs when infected individuals contaminate freshwater sources with faeces or urine containing parasite eggs. The eggs hatch, releasing miracidia into the water that penetrate the host snails and develop into infective cercariae. The cercariae are released from the snails and infect humans by penetrating the skin during contact with contaminated water [4]. Infection is often endemic in rural agricultural or fishing populations with poor sanitation [4]. Chronic infections cause significant morbidity including renal damage, anaemia, malnutrition, infertility and poor physical and cognitive development. Less frequently they can cause fatal complications from renal failure, portal hypertension and bladder cancer [4].

Praziquantel, a broad-spectrum anthelminthic, has been used for over 40 years as the cornerstone of schistosomiasis control, due to its safety, low cost and efficacy against all *Schistosoma* species [5, 6]. The World Health Organization (WHO) recommends that school-aged children in endemic areas are treated either annually (if prevalence is above 50% in school-aged children), every two years (if prevalence is between 10% and 49% in school-aged children), or upon entering and leaving primary school (if prevalence is lower than 10% in school-aged children) [5]. The WHO recommendations additionally suggest treating special groups of at-risk adults if schistosomiasis prevalence is above 10% in school-aged children, and entire communities if prevalence in school-aged children is above 50% [5, 7]. In the WHO’s landmark roadmap for NTD control, released in 2012, schistosomiasis control targets focus exclusively on school-aged children [8]. By 2020, the WHO target is that 75% of school-aged children at risk of schistosomiasis should be receiving regular praziquantel [8]. This target was set because the donations of praziquantel were sufficient to cover only this age group [9], and the WHO wanted to indicate an attainable goal for 2020. Globally, 68% of at-risk school-aged children received praziquantel in 2017, while coverage of at-risk adults was much lower at 16.9% [2].

With calls for elimination of schistosomiasis as a public health problem, defined by the WHO as less than 1% prevalence of heavy-intensity infections among school-aged children [7, 10], the effectiveness of treatment targeted to children has been called into question [11, 12]. It has been suggested that treatment should instead be delivered community-wide, to reduce disease prevalence and transmission to children from other vulnerable members in the community [12, 13], and minimise persistent untreated populations that can significantly impact on the success of control programs [14]. Results from mathematical modelling suggest that “mass” (community-wide) treatment is more effective for controlling schistosome infection than a “targeted” (children-only) program; however, this is dependent on local epidemiological settings, including pre-control burden in adults, school enrolment rates, and transmission intensity [14]. Additionally, although the outright cost of administering treatment to the entire community is higher than treating school-aged children only, modelling has shown community-wide drug administration to be a highly cost-effective strategy across different prevalence settings [15]. With many child-targeted programs being conducted through schools, community-wide treatment strategies would have the added benefit of increasing treatment coverage of non-enrolled school-aged children [16].

The Schistosomiasis Consortium for Operational Research and Evaluation (SCORE) has been established to answer strategic questions about schistosomiasis control and elimination. A number of trials have been implemented to assess the effectiveness of different control strategies in reducing the burden of active infection [17–21]. The results of these trials have been inconsistent, with some studies finding that community-wide treatment is more effective at reducing schistosome prevalence [22], whereas others report no significant difference between community-wide and child-targeted treatment [17, 20, 21].

To our knowledge, there is no systematic synthesis comparing mass and targeted delivery strategies (see Box 1) for schistosomiasis control in terms of their impact on schistosomiasis prevalence and intensity among children. This systematic review and meta-analysis aims to address this gap. Specific aims of this study are: (a) to summarise existing literature reporting the effects of mass and targeted praziquantel distribution on schistosomiasis prevalence and intensity in school-aged children, and (b) to examine the differential effect of mass and targeted praziquantel delivery on schistosomiasis prevalence and intensity in school-aged children.

**Box 1. Definitions of “mass” and “targeted” drug delivery in this paper**

Throughout this paper, we refer to “mass” and “targeted” drug delivery strategies, as defined by the World Health Organization:

**Mass drug administration**: the entire population of a given area is given anthelminthic drugs at regular intervals, irrespective of the individual infection status [23]. This is also known as community-wide treatment.

**Targeted drug administration**: specific risk groups in the population (defined by age, sex or other social characteristic) are treated at regular intervals, irrespective of infection status [23]. For the purposes of this paper, targeted drug administration refers to treatment targeted specifically to children.

## Methods

### Search strategy and selection criteria

This systematic review and meta-analysis was conducted according to PRISMA guidelines [24] (see S1 Checklist) and is available in PROSPERO, registration number CRD42018095377. Eligible studies were those that reported prevalence or intensity of schistosomiasis (any or all species) infection, before and after mass or targeted delivery of praziquantel. We included longitudinal studies as well as repeated cross-sectional studies in this review. Randomised controlled trials were included when randomisation was performed at the community or school level. There was no restriction on date or language of publication, geographical area, or length of study.

Studies were excluded if: treatment was delivered only to infected individuals; positive individuals were re-treated shortly after initial drug administration; all follow-up intervals (defined as the time between drug administration and examination) fell outside of a two month to 18-month timeframe; treatment intervals or follow-up times were not provided; or the drug administration strategy changed (from mass to targeted or vice versa) during the study with no interim data reported. Studies were excluded from quantitative analysis if they did not report initial and/or follow-up sample sizes.

We searched for studies through the databases MEDLINE, Embase and Web of Science on 15 February 2019. The following search terms were used: (a) disease-related terms: “Schistosomiasis” or “Bilharzia” or “*Schistosoma*” or “*Schistosoma mansoni*” or “*Schistosoma haematobium*” or “*Schistosoma japonicum*” or “*Schistosoma mekongi*” or “*Schistosoma guineensis*” or “*Schistosoma intercalatum*” or “Schistosome” or “Blood flukes” or “Trematode” or “*Trematoda*” or “Trematode infections” or “Trematode worms”, and (b) intervention-related terms: “Praziquantel” or “PZQ” or “Drug therapy” or “Chemotherapy” or “Preventive chemotherapy” or “Mass drug administration” or “Community based treatment” or “School based treatment”. The complete search strategy can be found in S1 Appendix. Additional studies were identified by hand-searching relevant review paper reference lists [14, 16, 25–31], monitoring the SCORE publication list, and contacting experts in the field.

Following de-duplication, studies were screened by title and abstract, followed by retrieval of full-text articles. All full-texts were then screened for eligibility against the study protocol. Articles published in English were examined by DMC and NEC. Articles published in languages other than English were screened by researchers fluent in those languages.

### Data extraction

Data were extracted from included studies by DMC and verified by NEC. Extracted data included initial and follow-up prevalence and intensity of the reported *Schistosoma* species; study location, design and duration; drug administration strategy (mass or targeted); drug administration platform (school-based, fixed community site or mobile community drug distributors); drug dose; number of treatment rounds; treatment interval(s); treatment coverage; diagnostic method; and interval between drug administration and follow-up.

Where studies reported multiple treatment arms with different treatment or follow-up intervals, data from each study arm were extracted. In studies with a control group that received drug treatment only and groups with additional interventions (e.g. health education), only data from the control group were extracted.

We contacted nine authors to request additional information, including aggregated follow-up prevalence and intensity data, follow-up interval, initial and follow-up sample sizes, praziquantel dose and interim prevalence data when delivery strategies changed during the study. Additional data on the number of infected individuals and intensity of infection were received from one study [21].

Study quality was assessed using a modified version of a validated scale designed to assess risk of bias in prevalence studies [32], as reported previously [26]. Studies were assessed against nine criteria encompassing internal and external validity.

### Statistical analysis

Analyses were performed using a similar methodology to our previously-reported meta-analysis comparing mass and targeted drug delivery strategies for soil-transmitted helminth infections [26]. All analyses were performed separately for each *Schistosoma* species.

To examine the impact of mass and targeted treatment on schistosome prevalence, an inverse variance weighted generalised linear model with robust error variances was used, in order to adjust for a number of key sources of heterogeneity. The covariates for the model were: drug administration strategy (mass or targeted); baseline prevalence; number of treatment rounds between baseline and follow-up; and follow-up interval (i.e., time between final treatment round and follow-up prevalence assessment). Treatment coverage was not included in the primary model because it was not reported in a large number of studies.

Where age-stratified prevalence was not available (two studies [33, 34]), infection prevalence in school-aged children was estimated from community prevalence using scaled age weights reported elsewhere [35]. The first reported assessment of infection prevalence and intensity was used as the baseline for all studies except one, where interim data were used as baseline because data were combined for study arms receiving treatment at different intervals for the first two years of the study [34]. Where multiple rounds of treatment were reported in one study, prevalence and intensity at the final follow-up were used. Where multiple follow-up intervals were reported, the closest to 12 months was used. Some studies were entered into the model multiple times to account for multiple species and strategies (mass or targeted) being reported [17, 21, 22, 36–41].

The outcome variable for the model was prevalence reduction (PReduc). This was defined as (p_1_-p_2_)/p_1_ = 1 –prevalence ratio, where p_1_ is the initial prevalence proportion and p_2_ is the post-treatment prevalence proportion. P_2_/p_1_ is the prevalence ratio (PRatio). As described previously, PReduc was truncated so that any increase between initial prevalence and follow-up prevalence would be reset to zero [26]. Coefficients were exponentiated to generate weighted odds ratios. Any study weights that were more than five times greater than the upper quartile were truncated and substituted with the threshold weight.

A secondary analysis was performed to pool prevalence reduction for each *Schistosoma* species. This was done by pooling PRatio, but results are reported as 1 –PRatio = PReduc (non-truncated). Results from each study were pooled using the inverse variance heterogeneity model [42]. Heterogeneity was assessed using Cochran’s Q and Higgins’ I^2^, with I^2^ greater than 50% indicating significant heterogeneity. Small-study effects were examined using Egger’s regression test (two-tailed p<0.1 indicating of asymmetry).

A number of sensitivity analyses were performed. Firstly, we added a covariate for reported treatment coverage (categorised as average of 75% coverage or greater across all treatment rounds; average of below 75% coverage across all treatment rounds; or treatment coverage not reported) to the generalised linear model. Secondly, we re-ran the generalised linear model excluding studies that reported greater than 50% initial prevalence, the cut-off for high-endemicity zones [5]. Finally, we re-ran the generalised linear model including follow-up prevalence measured after one treatment round (or closest), rather than after the final treatment round. Additionally, the generalised linear model and secondary meta-analysis were re-run using random effects model weights for comparison.

To compare intensity reduction between mass and targeted studies, given that limited data were available, simple box plots of egg reduction rates were created separately for mass and targeted studies. The box plots were weighted by sample size under the assumption that larger weights designate more accurately measured intensity reductions. Because there were insufficient studies to adjust for number of treatment rounds in this analysis, intensity reduction was calculated after one round of treatment (or closest) for consistency. Egg reduction rate was calculated using mean infection intensity at baseline and follow-up for each study, as follows: Egg reduction rate = (Mean intensity_baseline_−Mean intensity_followup_)/(Mean intensity_baseline_).

All meta-analyses were performed using MetaXL version 5.3 (Epigear International, Noosa, Australia). To run the generalised linear models and create the weighted box plots, Stata version 15.1 (StataCorp, College Station, TX, USA) was used.

## Results

**Fig 1:**
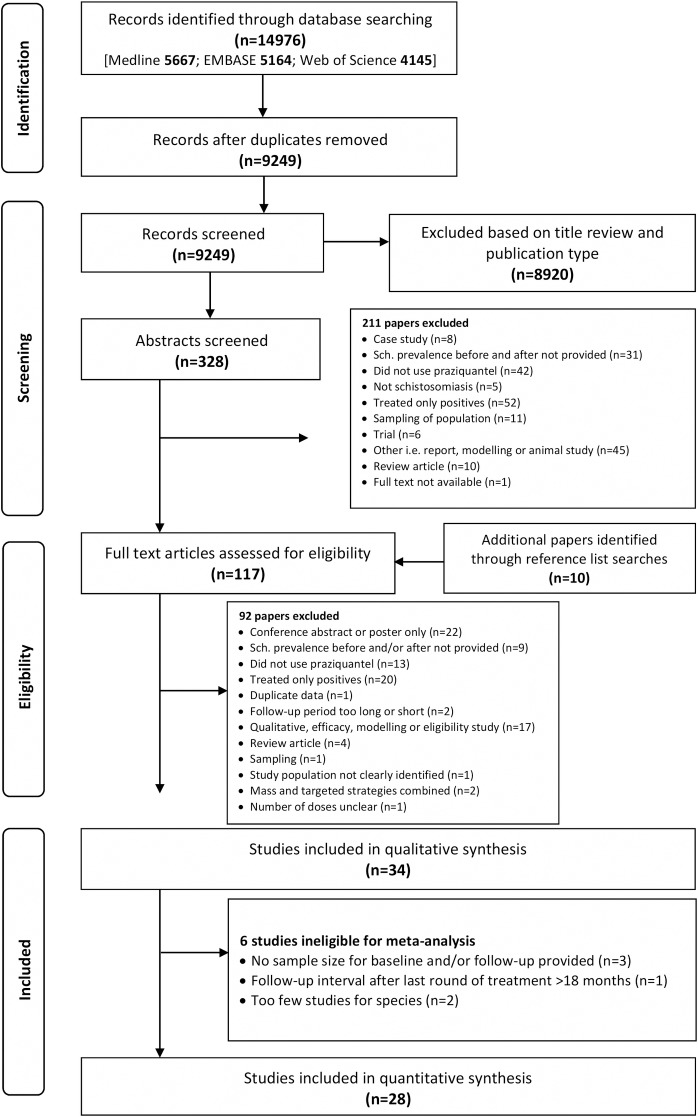
Process of selection of studies for inclusion in this synthesis.

After running the search terms and removing duplicate entries, 9,249 articles were considered for the systematic review process. An additional 10 studies were added through reference list searches. Thirty-four studies met the inclusion criteria for the systematic review. Of these, 28 studies were included for meta-analysis. The screening and selection process is illustrated in Fig 1. A summary of all included studies can be found in Table 1 and S1 Table.

**Table 1:** Characteristics of included studies for systematic review.

Author & year	Reference	Location	Drug delivery strategy	Treatment rounds prior to final follow-up	Follow-up interval after last treatment round	Study design	Drug delivery platform(s)
***Schistosoma mansoni***							
Abudho et al., 2018*	[43]	Kenya	Targeted	4 rounds, yearly	12 months	Repeated cross-sectional	School
Ahmed et al., 2012*	[44]	Sudan	Targeted	1 round	12 months	Longitudinal	School
Al Abaidani et al., 2016*	[45]	Oman	Mass	4 rounds, yearly	12 months	Repeated cross-sectional	Not reported
Assare et al., 2016*	[18]	Cote d’Ivoire	Targeted	1 round	11 months	RCT SCORE	School
Boisier et al., 1998*	[34]	Madagascar	Mass	4 rounds, yearly or 6 rounds, biannually (final round annual)	12 months	Longitudinal	Not reported
Hodges et al., 2012*	[46]	Sierra Leone	Targeted	1 round	6 months	Repeated cross-sectional	School
Kaatano et al., 2015	[47]	Tanzania	Mass	4 rounds, yearly	12 months	Repeated cross-sectional	School, fixed community site, mobile CDDs
Karanja et al., 2017*	[19]	Kenya	Targeted	2 or 4 rounds, yearly or biennially	12, 24 and 36 months	RCT SCORE	School
Mwinzi et al., 2012*	[48]	Kenya	Mass	1 round	6 months	Repeated cross-sectional	Mobile CDDs
Olsen et al., 2018*	[21]	Tanzania	Both	2 or 4 rounds, yearly or biennially	12, 24 and 36 months	RCT SCORE	School, fixed community site
Onkanga et al., 2016	[20]	Kenya	Both	2 rounds, yearly	12 months	RCT SCORE	School, mobile CDDs
Wanjala et al., 2013*	[49]	Kenya	Targeted	1 round	18 months	Longitudinal	School
Zhang et al., 2007*	[22]	Uganda	Botb	2 rounds, yearly	12 months	Longitudinal	School, mobile CDDs
**Both *Schistosoma mansoni* and *Schistosoma haematobium***							
Brinkmann et al., 1988*	[36]	Mali	Both	1 round	12 months	Repeated cross-sectional	Not reported
Koukounari et al., 2007*	[37]	Burkina Faso	Targeted	1 round	12 months	Longitudinal	School, fixed community site, mobile CDDs
Landoure et al., 2012*	[38]	Mali	Mass	3 or 4 rounds, yearly (with 1 year break)	12 and 24 months	Repeated cross-sectional	School, mobile CDDs
Massa et al., 2009*	[39]	Tanzania	Targeted	1 round	12 months	Repeated cross-sectional	School, mobile CDDs
Mwandawiro et al., 2019*	[40]	Kenya	Targeted	2 or 4 rounds, yearly	12 months	Repeated cross-sectional	School
Ouedraogo et al., 2016	[50]	Burkina Faso	Targeted	4 rounds, approximately biennially	12 or 24 months	Repeated cross-sectional	School, fixed community site, mobile CDDs
Toure et al., 2008*	[41]	Burkina Faso	Targeted	1 round	12 and 24 months	Longitudinal	School, fixed community site, mobile CDDs
***Schistosoma haematobium***							
Adewale et al., 2018*	[51]	Nigeria	Targeted	1 round	12, 24, 36, 48 and 60 months	Longitudinal	School
Chaula & Tarimo, 2014*	[52]	Tanzania	Targeted	2 rounds, yearly	12 months	Longitudinal	School
Garba et al., 2004*	[33]	Niger	Mass	1 round	10 and 28 months	Repeated cross-sectional	Not reported
Hopkins et al., 2002	[53]	Nigeria	Mass	2 rounds, yearly	12 months	Repeated cross-sectional	Mobile CDDs
Janitschke et al., 1989*	[54]	Yemen	Mass	1 round	12 months	Repeated cross-sectional	Not reported
Mduluza et al., 2001*	[55]	Zimbabwe	Targeted	7 rounds, bi-monthly	2 months	Longitudinal	Not reported
N’Goran et al., 2001*	[56]	Cote d’Ivoire	Targeted	1 round or 2 rounds, yearly	6, 12, 18 and 24 months	Longitudinal	School
Pennance et al., 2016*	[57]	Tanzania	Mass	4 rounds, biannually	7–8 months	Repeated cross-sectional	School, mobile CDDs
Phillips et al., 2017*	[17]	Mozambique	Both	2 or 4 rounds, yearly or biennially	12, 24 and 36 months	RCT SCORE	School, fixed community site, mobile CDDs
Saathoff et al., 2004*	[58]	South Africa	Targeted	1 round	3, 16, 41 and 53 weeks	Longitudinal	School
Shehata et al., 2018*	[59]	Zambia	Targeted	1 round	6 and 12 months	Longitudinal	School
Stothard et al., 2009*	[60]	Tanzania	Targeted	2 rounds, yearly	12 months	Repeated cross-sectional	School
***Schistosoma japonicum***							
Lin et al., 1997	[61]	China	Mass	2 rounds, yearly	12 months	Longitudinal	Fixed community site
Zhang et al., 1998	[62]	China	Mass	1 round	12 months	Longitudinal	Not reported

RCT = randomised controlled trial, SCORE = study conducted by Schistosomiasis Consortium for Operational Research and Evaluation, CDD = community drug distributor

*Included in meta-analysis

### Characteristics of included studies

Of the 34 included studies, 13 (38.2%) reported on only *S*. *mansoni*, 12 (35.3%) reported on only *S*. *haematobium*, seven (20.6%) reported on both *S*. *mansoni* and *S*. *haematobium*, and two (5.9%) reported on *S*. *japonicum* (Table 2). For *S*. *mansoni*, nine of 20 studies (45.0%) used a mass drug administration strategy and 14 (70.0%) used a targeted strategy. Three of these studies (15.0%) reported both strategies. For *S*. *haematobium*, seven of 19 studies (36.8%) used a mass drug administration strategy and 14 (73.7%) used a targeted strategy. Two of these studies (10.5%) reported both strategies. Of the five studies that included both mass and targeted strategies, three of these were randomised controlled trials that compared mass and targeted treatment arms within the study [17, 20, 21], one study used baseline village prevalence to direct mass or targeted treatment [36], and one study presented results for individual village groups that adopted mass or targeted strategies [22]. Both studies of *S*. *japonicum* reported mass drug administration.

**Table 2:** Descriptive indicators separated by schistosome species.

	*S*. *mansoni* (n = 20*)		*S*. *haematobium* (n = 19*)		*S*. *japonicum* (n = 2)
	Mass	Targeted	Mass	Targeted	Mass
**Drug delivery strategy**	9 (45%)^	14 (70%)^	7 (37%)^	14 (74%)^	2 (100%)
**Delivery platform#**					
School	2 (22%)	14 (100%)	2 (29%)	12 (86%)	-
Community–fixed site	2 (22%)	3 (21%)	1 (14%)	3 (21%)	1 (50%)
Community–mobile unit	5 (56%)	4 (29%)	4 (57%)	4 (29%)	-
Not reported	3 (33%)	-	3 (43%)	2 (14%)	1 (50%)
**Number of treatment rounds†**					
1 round	3 (33%)	8 (57%)	3 (43%)	8 (57%)	2 (100%)
Multiple rounds	7 (78%)	7 (50%)	5 (71%)	8 (57%)	-
**Follow up after final treatment round†**					
Less than 12 months	1 (11%)	2 (14%)	2 (29%)	6 (43%)	-
12 months	8 (89%)	11 (79%)	4 (57%)	13 (93%)	2 (100%)
Greater than 12 months	2 (22%)	6 (43%)	4 (57%)	9 (64%)	-

* These totals include seven studies that report both *S*. *mansoni* and *S*. *haematobium*.

^ Three studies for *S*. *mansoni* and two studies for *S*. *haematobium* reported both mass and targeted strategies

# Numbers add to greater than 100% because studies used more than one delivery platform.

**†** Number of treatment rounds and follow up after final treatment round may add to greater than 100% as this incorporates studies that have multiple treatment arms with differing values for these variables.

As shown in Table 2, studies of targeted drug distribution primarily used a school-based platform to treat school-aged children. Some studies additionally used other platforms to reach non-enrolled school-aged children, including fixed community sites at dispensary units and mobile community drug distributors. One study compared school-based and community-based drug delivery for school-aged children [39], and one study treated only preschool children, aged two to six years [55]. Two studies did not report delivery platform [36, 55]. Studies of mass drug distribution primarily conducted drug administration through mobile community drug distributors. Fixed community sites and schools were also used to distribute drugs to the entire community. Six studies did not report the delivery platform [33, 34, 36, 45, 54, 62].

The number of treatment rounds varied from one to seven rounds, with treatment intervals ranging from two to 24 months. The follow-up interval after the final treatment round ranged from three weeks to 60 months. The most common follow-up interval after the final treatment round for all three species and both drug administration strategies was 12 months (27 studies, 79.4%).

Nine potential deficiencies were assessed in terms of risk of bias (S2 Table). The most common deficiencies were a response rate of less than 75% (or not reported) in 31 studies and praziquantel not delivered to at least 75% of the population (or not reported) in 19 studies. All other deficiencies were less common.

All 22 studies of *S*. *mansoni* and *S*. *japonicum* used stool samples to determine infection prevalence and intensity; all used the Kato-Katz diagnostic method. All 19 *S*. *haematobium* studies used urine samples to determine infection prevalence and intensity. Seventeen studies (89.5%) used the urine filtration method and four studies (21.1%) used urine dipsticks; two of these studies (10.5%) used both methods.

As shown in S1 Table, the most common additional medication was albendazole (11 studies, 32.4%), with mebendazole (two studies, 5.9%) and ivermectin (one study, 2.9%) also administered. Twenty-one studies (61.8%) reported no additional medications. Health education (e.g. videos, posters, broadcasts, reading material) was reported in seven studies (20.6%). Snail control using tilapia fish or molluscicide (two studies, 5.9%) and water source improvements with pumped wells (one study, 2.9%) were also reported. Twenty-seven studies (79.4%) reported no additional interventions apart from drug delivery.

Most studies were conducted in Africa (30 studies, 88.2%), with two studies (5.9%) in the Middle East and two studies in Asia. Tanzania and Kenya (six studies (17.6%) each) were the most commonly studied countries. Fourteen studies (41.2%) used a longitudinal design, while 15 (44.1%) were repeated cross-sectional studies and five were randomised controlled trials conducted by SCORE.

Treatment coverage was reported in 20 of 34 studies (58.8%), ranging from 22.7% to 129.8% (see S1 Table). Coverage values over 100% were seen due to inaccurate estimates of population size. Sixteen of these studies (80.0%) reported coverage greater than 75% for at least one treatment round.

Schistosome prevalence was reported in all included studies, while a measure of infection intensity was reported in 30 of the 34 studies (88.2%). Four of these studies reported only the proportion of high intensity infections, and two studies reported intensity only at baseline.

### Quantitative analysis

Only *S*. *mansoni* and *S*. *haematobium* had sufficient studies to perform quantitative analysis. Results from the inverse variance weighted generalised linear model are shown in Table 3.

**Table 3:** Odds ratio of prevalence reduction* for selected covariates, stratified by *Schistosoma* species (inverse variance weighted generalised linear model with robust error variance).

	Odds ratio (95% CI)	p-value	R^2^
***Schistosoma mansoni***			
Mass (n = 7) vs targeted (n = 12) treatment	0.47 (0.13–1.68)	0.227	0.126
Baseline prevalence (%)	1.02 (0.99–1.05)	0.292	
Number of treatment rounds	0.95 (0.64–1.42)	0.786	
Follow-up time (months)	1.06 (0.92–1.23)	0.390	
***Schistosoma haematobium***			
Mass (n = 6) vs targeted (n = 13) treatment	0.41 (0.06–3.03)	0.358	0.279
Baseline prevalence (%)	0.99 (0.95–1.05)	0.926	
Number of treatment rounds	0.76 (0.49–1.20)	0.219	
Follow-up time (months)	1.48 (0.50–4.40)	0.452	

* Prevalence reduction = (Prevalence_follow-up_−Prevalence_baseline_) / Prevalence_baseline_, truncated such that any prevalence increase was reset to zero

For *S*. *mansoni*, 19 studies were included in the model, with two studies including two treatment arms [21, 22]. There was no significant difference in prevalence reduction following mass versus targeted treatment (odds ratio (OR) 0.47, 95% confidence interval (CI) 0.13–1.68, p = 0.227). Number of treatment rounds, baseline prevalence and follow-up time were not significantly associated with prevalence reduction.

Similarly, for *S*. *haematobium*, 19 studies were included in the model, with two studies including two treatment arms [17, 36]. There was no significant difference between mass and targeted drug treatment (OR 0.41, 95% CI 0.06–3.03, p = 0.358). Baseline prevalence, number of treatment rounds, and follow-up time were not significantly associated with prevalence reduction.

**Fig 2:**
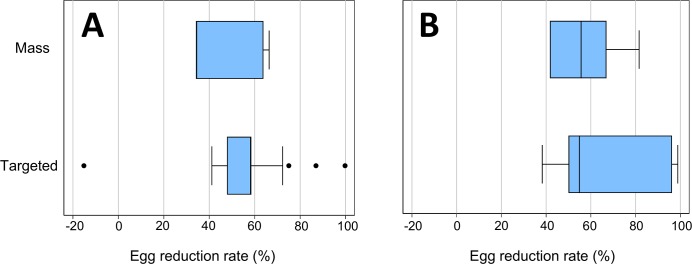
Boxplots of infection intensity reduction for *S*. *mansoni* (A) and *S*. *haematobium* (B) for studies using mass and targeted strategies. Studies were weighted according to their sample size.

Results for intensity reduction are presented in Fig 2. There are no apparent differences in median egg reduction rates between mass and targeted praziquantel delivery for either *S*. *mansoni* or *S*. *haematobium*.

In sensitivity analyses, adding a covariate for treatment coverage did not significantly affect the results of the generalised linear model (S3 Table). Furthermore, results remained generally robust when high-prevalence studies were excluded and when follow-up prevalence was measured after one treatment round or closest (S4 Table). For *S*. *haematobium*, when studies with greater than 50% baseline prevalence were excluded, only a very small number of studies remained, leading the model to become unstable with very wide confidence intervals.

Results of the secondary meta-analyses synthesising the non-truncated prevalence reduction estimates from individual studies are shown in Table 4. Pooled prevalence reduction is shown separately for mass and targeted studies, stratified by number of treatment rounds. There was significant heterogeneity among included studies. In targeted studies, I^2^ was 96.7% for *S*. *mansoni* and 98.5% for *S*. *haematobium*. In mass studies, I^2^ was 93.3% for *S*. *mansoni* and 98.6% for *S*. *haematobium*.

**Table 4:** Meta-analysis results showing pooled prevalence reduction estimates (non-truncated), shown for mass and targeted studies for each schistosome species, stratified by number of treatment rounds.

Delivery method	Number of treatment rounds	PReduc* (95% CI)	Cochran's Q	*p* value (Cochran’s Q)	Number of study datasets
***Schistosoma mansoni***					
Mass	One round	0.22 (-0.76–0.66)	27.63	<0.001	4
	Multiple rounds	0.34 (0.07–0.53)	36.55	<0.001	3
Targeted	One round	0.41 (0.19–0.57)	84.34	<0.001	7
	Multiple rounds	0.33 (-0.33–0.67)	235.49	<0.001	5
***Schistosoma haematobium***					
Mass	One round	0.57 (0.24–0.75)	46.86	<0.001	3
	Multiple rounds	0.34 (-0.04–0.58)	111.41	<0.001	3
Targeted	One round	0.69 (0.45–0.82)	216.34	<0.001	7
	Multiple rounds	0.60 (-0.01–0.84)	574.68	<0.001	6

*PReduc = (Prevalence_follow-up_−Prevalence_baseline_) / Prevalence_baseline_

The results of analyses conducted using random effects weights are depicted in S5 Table and S6 Table. Re-analysis using this approach did not significantly affect study results. In terms of small study effects, Egger’s regression showed evidence of mild funnel plot asymmetry for *S*. *mansoni* (intercept -4.10, p = 0.022), but not for *S*. *haematobium* (intercept -3.03, p = 0.335).

## Discussion

Treatment of schistosomiasis has been scaled up over the last decade from test-and-treat strategies towards large-scale preventive drug administration programs. Recent modelling studies predict community-wide treatment programs to be the most effective strategy for controlling infection [14, 15]. To our knowledge, this systematic review and meta-analysis is the first synthesis of its kind, comparing the effects of mass and targeted delivery strategies on schistosomiasis prevalence and intensity in school-aged children.

The results of this analysis show that based on currently published studies, there is no detectable difference between mass and targeted drug administration strategies on the reduction in *S*. *mansoni* or *S*. *haematobium* infection prevalence or intensity in school-aged children. This does not align with our hypothesis, which was supported by previously-published mathematical modelling predictions [14] and a similar meta-analysis on soil-transmitted helminths [26]. The current analysis reflects only the findings of the 28 studies that were considered, including recent cluster-randomised controlled trials conducted by the SCORE initiative that found community-wide and child-targeted treatment equally effective in reducing prevalence in *S*. *mansoni* and *S*. *haematobium* infections [17, 20, 21]. An earlier study that applied either mass or targeted treatment to different villages also found similar prevalence reductions following mass and targeted drug delivery for *S*. *haematobium* [36].

One of the most plausible reasons for these results is insufficient treatment coverage in mass drug administration programs. Compared to child-targeted programs conducted through schools, mass drug administration programs may face more challenges in reaching a majority of the population eligible for treatment. Many studies included in our review did not report treatment coverage, and among those that did, reported coverage varied widely between studies, and was often inconsistent across treatment rounds within studies. Due to the large amount of studies that did not report treatment coverage, we were unable to adjust for this in our primary analysis. However, our findings for mass versus targeted treatment did not change when we conducted a sensitivity analysis controlling for treatment coverage. Further issues with reported treatment coverage are that underestimation of the target population is common and leads to an overestimation of treatment coverage [20], and also that there may be considerable discrepancies between reported treatment coverage (those who receive tablets) and treatment compliance (those who actually ingest the tablets) [63]. Compliance is an important consideration when reporting treatment coverage; however, there is considerable heterogeneity in the defining and reporting of treatment compliance in existing literature [63].

Another potential explanation for our findings is that in some settings, school-aged children may play a dominant role in driving transmission. Infections peak in childhood, leading school-aged children to generally carry more infection than other community members [4]. Therefore, in some settings, expanding treatment programs to other community members may not have a detectable effect on transmission among school-aged children. Furthermore, it is known that in many settings, there are hotspots of infection that have a disproportionate influence on driving transmission [64]. These persistent low-prevalence populations tend to remain, regardless of the drug administration strategy or how well drug administration programs are implemented [57]. Such hotspots may explain why significant differences between mass and targeted treatment were not evident in this review.

Several limitations in this study must be acknowledged. Firstly, only a relatively small number of studies were included in meta-analysis, especially for mass treatment. Secondly, there is potential for confounding from factors we were unable to include in our model, such as water, sanitation and hygiene (WASH) conditions, socioeconomic status and treatment coverage. An important potential confounder is the proximity of the infected population to local contaminated bodies of water [22], and the frequency and duration of water contact, especially in children [18]. Several studies have shown that populations living closer to local water bodies are more likely to have a higher prevalence and rate of reinfection [19, 22, 65]. Thirdly, limitations of existing diagnostic techniques for identifying schistosome infections may have affected our findings. Both the Kato-Katz technique (used to diagnose for *S*. *mansoni* and *S*. *japonicum* infections) and urine filtration (used for *S*. *haematobium*); are known to have low sensitivity, particularly for detecting light-intensity infections in areas with low endemicity [66, 67]. This is a source of measurement error that can underestimate the actual prevalence of infection in a population, creating a non-differential misclassification and potentially biasing findings towards the null hypothesis. Finally, two included studies did not have age-stratified data available, so we used standardised weights for calculating prevalence in school-aged children. These weights were taken from a study published in 1998 [35]. The age distribution and schistosome prevalence may vary between communities and prevalence reduction among school-aged children may not reflect that of other age groups.

Our review focuses on the impact of mass and targeted drug delivery strategies on school-aged children, who are recognised as the group at highest risk of schistosomiasis-associated morbidity. However, it is important to note that expanding control programs community-wide would provide the advantage of reducing morbidity in other age groups (i.e., preschool-aged children, adolescents, and adults), in whom the burden of schistosomiasis-associated morbidity may also be significant [68, 69]. WHO guidelines currently do not recommend a baseline assessment of infection prevalence and intensity in age groups other than school-aged children [70]. However, recent mathematical modelling highlights that including a broader range of age groups in baseline assessments, as well as ongoing monitoring, is important in order to determine appropriate control strategies for defined regions [71].

Although current targets, drug donations, and operational guidelines for schistosomiasis control focus on school-aged children [8, 9, 23], the burden of schistosomiasis among both adults and preschool-aged children has been acknowledged [5, 72]. At-risk adults are recommended to receive regular praziquantel in certain epidemiological settings [5], although global coverage remains low [2]. On the other hand, preschool-aged children are not included in WHO guidelines for schistosomiasis control because the safety of praziquantel in children under 4 years of age has not been established, and because there is no suitable paediatric formulation available [72, 73]. A recent dose-ranging study identified that a single 40mg/kg dose of praziquantel can be administered safely and efficaciously in children under 5 years of age [73], and a paediatric formulation of praziquantel is currently in development [74]. Treatment of preschool-aged children could be achieved by utilising existing platforms, such as child health days, as is done for other NTDs [75], if this formulation was made available free of charge.

Our findings highlight the importance of additional strategies beyond regular drug administration in achieving community-wide control of schistosomiasis. Praziquantel remains effective at treating *S*. *mansoni* and *S*. *haematobium* [76], but due to ongoing environmental reservoirs of disease, it does not stop reinfection. Rapid reinfection can occur in endemic areas [77], indicating that treatment should be accompanied with WASH interventions to improve water and sanitation conditions and hygiene behaviours. These interventions may include provision of a safe water supply, education to end open defecation, and safe contact with contaminated bodies of water [78]. As schistosomiasis is transmitted to humans through contact with contaminated freshwater snails, and amplification of parasite numbers occurs within the intermediate host [4], snail control with molluscicide or similar treatment should also be considered [79]. Only a very small number of studies in this review included such strategies.

In conclusion, although our analysis of current literature does not agree with mathematical modelling findings, there are limitations to existing studies and to this meta-analysis. There are further cluster-randomised controlled trials comparing strategies in development (C. King and A. Amadou, personal communication), which will provide more information on the effect of mass versus targeted treatment. Despite the findings presented here, it is nonetheless likely that mass treatment–when delivered with high coverage rates–will be more beneficial in some settings than targeted treatment for reducing infections among school-aged children. More research is needed to address issues with achieving coverage targets, implementation of WASH improvements, and addressing disease hotspots and sources of rapid reinfection. Consideration of these factors will assist with optimising control programs in the push towards eliminating schistosomiasis as a public health problem.

## Supporting information

S1 Checklist Completed PRISMA checklist. (DOC)

S1 Appendix Search strategy. (DOCX)

S1 Table Additional characteristics of included studies. (DOCX)

S2 Table Quality assessment of included studies. (DOCX)

S3 Table Odds ratio of prevalence reduction for selected covariates, including treatment coverage, stratified by *Schistosoma* species (inverse variance weighted generalised linear model with robust error variance). (DOCX)

S4 Table Odds ratio of prevalence reduction for selected covariates in sensitivity analysis, stratified by *Schistosoma* species (inverse variance weighted generalised linear model with robust error variance). (DOCX)

S5 Table Odds ratio of prevalence reduction for selected covariates, stratified by *Schistosoma* species (random effects weighted generalised linear model with robust error variance). (DOCX)

S6 Table Meta-analysis results showing pooled prevalence reduction estimates (non-truncated) using random effects weights. (DOCX)
