# Supplementary material for: Differential impact of mass and targeted praziquantel delivery on schistosomiasis control in school-aged children: A systematic review and meta-analysis
Source: PLoS Negl Trop Dis. 2019 Oct 11;13(10):e0007808. doi: 10.1371/journal.pntd.0007808 (PMC6808504; doi:10.1371/journal.pntd.0007808)
Supplement: S1 Appendix — (DOCX) [file pntd.0007808.s002.docx]

**S1 Appendix. Search strategy**

1. Schistosomiasis
2. Bilharzia
3. Schistosoma
4. Schistosoma mansoni
5. Schistosoma haematobium
6. Schistosoma japonicum
7. Schistosoma mekongi
8. Schistosoma guineensis
9. Schistosoma intercalatum
10. Schistosome
11. Blood flukes
12. Trematode
13. Trematoda
14. Trematode infections
15. Trematode worms
16. (1 or 2 or 3 or 4 or 5 or 6 or 7 or 8 or 9 or 10 or 11 or 12 or 13 or 14 or 15)
17. Praziquantel
18. PZQ
19. Drug therapy
20. Chemotherapy
21. Preventive chemotherapy
22. Mass drug administration
23. Community based treatment
24. School based treatment
25. (17 or 18 or 19 or 20 or 21 or 22 or 23 or 24)
26. 16 and 25
