# Supplementary material for: Differential impact of mass and targeted praziquantel delivery on schistosomiasis control in school-aged children: A systematic review and meta-analysis
Source: PLoS Negl Trop Dis. 2019 Oct 11;13(10):e0007808. doi: 10.1371/journal.pntd.0007808 (PMC6808504; doi:10.1371/journal.pntd.0007808)
Supplement: S1 Table — (DOCX) [file pntd.0007808.s003.docx]

**S1 Table. Additional characteristics of included studies**

| Author & year | Diagnostic method | Other medications and interventions | Baseline prevalence, % (N) | Follow up prevalence, % (N) | Mean infection intensity at baseline | Mean infection intensity at follow-up | Treatment coverage | Duration of study |
| --- | --- | --- | --- | --- | --- | --- | --- | --- |
| *Schistosoma mansoni* | | | | | | | | |
| Abudho et al., 2018* | Kato-Katz | ALB | 44.7% (1110) | 14% (1440) | 90.4epg | 8.1epg | >90% | 4 years |
| Ahmed et al., 2012* | Kato-Katz | ALB | 59.1% (2741) | 37.4% (2521) | 116.7epg (GM) | 68.7epg (GM) | Not reported | 1 year |
| Al Abaidani et al., 2016* | Kato-Katz,  serology | Snail control, health education | 9.1% (165) | 0% (109) | Not reported | Not reported | Not reported | 7 years |
| Assare et al., 2016* | Kato-Katz | Not reported | 22.1% (7011) | 12.8% (4667) | 94.9epg (AM) | 109.3epg (AM) | 84.2% | 5 years (1^st^ year data) |
| Boisier et al., 1998* | Kato-Katz | Not reported | 9.9% (289) | 19.3% (289) | 69epg (GM) | 27epg GM) | Not reported | 3 years |
| Hodges et al., 2012* | Kato-Katz | MEB | 69% (515) | 38.2% (448) | 170.8epg | 47.3epg | 94% | 6 months |
| Kaatano et al., 2015 | Kato-Katz | ALB, water, health education | 42.2% (no sample size) | 6.7% (no sample size) | 181.2epg | 92.7epg | 42.6-76.8% | 5 years |
| Karanja et al., 2017* | Kato-Katz | Not reported | 17.7% (4614) | 8.7% (7206) | 15.9epg (AM) | 8.7epg (AM) | 93.9% | 5 years |
| Mwinzi et al., 2012* | Kato-Katz | ALB | 17.8% (492) | 11.8% (576) | 166.8epg | 60.3epg | 54.2-96.6%, average 77.5% | 6 months |
| Olsen et al., 2018* | Kato-Katz | Not reported | Mass 56.9% (1384)  Targeted 52.5% (1309) | Mass 40% (942)  Targeted 42% (1033) | Mass 151epg (AM)  Targeted 112.5epg (AM) | Mass 40.8epg (AM)  Targeted 43.7epg (AM) | CWT 76.7-79.4%  SBT 76.9-82.6% | 5 years |
| Onkanga et al., 2016 | Kato-Katz | Not reported | Mass 60% (no SS)  Targeted 63% (no SS) | Mass 48% (no SS)  Targeted 34% (no SS) | Mass 88.5epg (AM)  Targeted 91.6epg (AM) | Mass 76.9epg (AM)  Targeted 31.8epg (AM) | CWT 84.1-87.6%  SBT 84.6-89.8% | 5 years (2^nd^ year data) |
| Wanjala et al., 2013* | Kato-Katz | Health education | 32.1% (972) | 11% (972) | 90epg (GM) | Not reported | Not reported | 2 years |
| Zhang et al., 2007* | Kato-Katz | ALB, health education | Mass 77.2% (620)  Targeted 29.9% (795) | Mass 38.5% (620)  Targeted 8% (795) | Mass 522.3epg (AM)  Targeted 60.2epg (AM) | Mass 86.6epg (AM)  Targeted 12.7epg (AM) | >75% | 2 years |
| Both *Schistosoma mansoni* and *Schistosoma haematobium* | | | | | | | | |
| Brinkmann et al., 1988* | Kato-Katz, urine filtration | Not reported | SH Mass 65.3% (11078)  SH Targeted 24.5% (760)  SM Mass 49% (7175) | SH Mass 10.3% (9306)  SH Targeted 10.4% (1046)  SM Mass 37.7% (5475) | Not reported | Not reported | Not reported | 3 years |
| Koukounari et al., 2007* | Kato-Katz, urine filtration | ALB | SM 6.2% (1727)  SH 53.9% (1727) | SM 0.2% (1131)  SH 5.8% (1131) | SM 8.0epg (AM)  SH 83.6ep10ml (AM) | SM 0.02epg (AM)  SH 0.9ep10ml (AM) | Not reported | 1 year |
| Landoure et al., 2012* | Kato-Katz, urine filtration | Not reported | SM 17.3% (648)  SH 88% (648) | SM 12.7% (640)  SH 61.7% (640) | SM 88.2epg (AM)  SH 180.4ap10ml (AM) | SM 43.2epg (AM)  SH 33.2aep10ml | 56.4-75.1% | 6 years |
| Massa et al., 2009* | Kato-Katz, urine filtration | ALB | SM 28.1% (1140)  SH 26.4% (1140) | SM 9.8% (1143)  SH 13.5% (1143) | SM 64epg (AM)  SH 2ep10ml (AM) | SM 16epg (AM)  SH 1ep10ml (AM) | 80.3-82.1% | 1 year |
| Mwandawiro et al., 2019* | Kato-Katz, urine filtration | ALB | SM 2.1% (21432)  SH 14.8% (21432) | SM 1.7% (20941)  SH 2.4% (20941) | SM 12epg  SH 16epg | SM 5epg  SH 2epg | 65.6-104% | 5 years |
| Ouedraogo et al., 2016 | Kato-Katz, urine filtration | Not reported | SH 55.8% (1644)  SM Not reported | SH 7.5% (1280)  SM 1.2% (3514) | SH 91.3ep10ml (AM) | SH 9.4ep10ml (AM)  SM 1.0epg (AM) | Reported per village | 9 years |
| Toure et al., 2008* | Kato-Katz, urine filtration | ALB | SH 59.6% (763)  SM 2.8% (322) | SH 6.2% (763)  SM 1.6% (763) | SH 94.2ep10ml (AM)  SM 4.6epg (AM) | SH 6.8ep10ml (AM)  SM 0.6epg (AM) | >90% | 2 years |
| *Schistosoma haematobium* | | | | | | | | |
| Adewale et al., 2018* | Urine filtration | Not reported | 24.9% (434) | 7.7% (415) | 20.5ep10ml moderate  4.4ep10ml heavy | 7.7ep10ml moderate  0ep10ml heavy | Not reported | 1 year |
| Chaula & Tarimo, 2014* | Urine filtration | Water and snail education | 26% (488) | 15% (488) | Not reported | 9.6% high  12.3% med  78.1% low | 39.5-43.6% | 2 years |
| Garba et al., 2004* | Urine filtration | Not reported | 74.1% (232) | 39.8% (201) | 9.9% heavy | 6.4% heavy | 69.9-78.2% | 3 years |
| Hopkins et al., 2002 | Dipstick reagent test | IVM, ALB | 50% (no SS)  83.3% (no SS) | 3% (226)  5% (240) | Not reported | Not reported | 84.8% | 2 years |
| Janitschke et al., 1989* | Urine filtration, dipstick reagent test | Health education, snail control | 36.3% (441) | 5.1% (472) | Not reported | Not reported | Not reported | 1 year |
| Mduluza et al., 2001* | Urine filtration | Not reported | 51.8% (595) | 1.2% (246) | 110ep10ml | 2ep10ml | Not reported | 14 months |
| N’Goran et al., 2001* | Urine filtration | Not reported | 87.6% (685) | 33% (174) | 14.2ep10ml (GM) | 2.6ep10ml (GM) 1 round  3.7ep10ml (GM) 2 rounds | Not reported | 2 years |
| Pennance et al., 2016* | Urine filtration | Not reported | 10.3% (680) | 16.8% (744) | 5ep10ml | Not reported | 67.2-92.7% | 2 years |
| Phillips et al., 2017* | Dipstick reagent test, urine filtration | Not reported | 66.7% (7229) | 42.5% (13626) | 69.1ep10ml (AM) | 58.1ep10ml (AM) | 22.7-129.8% | 5 years |
| Saathoff et al., 2004* | Urine filtration | ALB | 68.3% (1109) | 20.1% (825) | 16.1ep10ml (GM) | 0.6ep10ml (GM) | 76.8% | 1 year |
| Shehata et al., 2018* | Urine filtration | Not reported | 28.6% (975) | 20.3% (310) | 84.9% light  15.1% heavy | 43% light  20% heavy | Not reported | 4 years |
| Stothard et al., 2009* | Dipstick reagent test | ALB | 13.2% (2002) | 6.4% (3993) | 0.5ep10ml (GM) | 0.2ep10ml (GM) | Not reported | 3 years |
| *Schistosoma japonicum* | | | | | | | | |
| Lin et al., 1997 | Kato-Katz | Health education | 26% (235) | 10.7% (290) | 1.9epg | 0.6epg | Not reported | 2 years |
| Zhang et al., 1998 | Kato-Katz | Not reported | 18.2% (680) | 5.2% (538) | 363.6epg (AM)  113.6epg (GM) | 87.3epg (AM)  45.6epg (GM) | 76.0% | 1 year |

ALB = albendazole; IVM = ivermectin; MEB = mebendazole; SH = *Schistosoma haematobium*; SM = *Schistosoma mansoni*; SS = sample size; AM = arithmetic mean; GM = geometric mean; epg = eggs per gram of faeces; ep10ml = eggs per 10mL of urine; CWT = community-wide treatment; SBT = school-based treatment

*Studies included in meta-analysis
