# Supplementary material for: Differential impact of mass and targeted praziquantel delivery on schistosomiasis control in school-aged children: A systematic review and meta-analysis
Source: PLoS Negl Trop Dis. 2019 Oct 11;13(10):e0007808. doi: 10.1371/journal.pntd.0007808 (PMC6808504; doi:10.1371/journal.pntd.0007808)
Supplement: S2 Table — (DOCX) [file pntd.0007808.s004.docx]

**S2 Table. Quality assessment of including studies**

| Author & year | External validity |  |  |  | Internal validity |  |  |  |  |
| --- | --- | --- | --- | --- | --- | --- | --- | --- | --- |
|  | The target population was clearly described and was a close representation of the general population in relation to relevant variables, e.g. age and sex | The sampling frame was clearly described and was a true or close representation of the target population | Some form of random selection was used to select the sample, or a census was undertaken | Response rate was ≥75%, or analysis showed no significant difference in relevant demographic characteristics between responders and nonresponders | The schistosome detection method was clearly described and was reliable and valid | Same method of schistosome diagnosis was used for all subjects in the study | Subjects were selected or recruited from the same or similar populations at both time points, or the same cohort was follow longitudinally | The sampling method was well described and the same method was used at both time points, or the same cohort was followed longitudinally | The distribution strategy of schistosome medication was clearly described and delivered to at least 75% of the target population |
| Abudho et al., 2018* | Y | Y | Y | N | Y | Y | Y | N | Y |
| Adewale et al., 2018* | Y | Y | Y | Y | Y | Y | Y | Y | N |
| Ahmed et al., 2012* | Y | Y | Y | N | Y | Y | Y | Y | N |
| Al Abaidani et al., 2016* | Y | Y | Y | N | Y | N | Y | N | N |
| Assare et al., 2016* | Y | Y | Y | N | Y | Y | Y | Y | Y |
| Boisier et al., 1998* | Y | Y | Y | N | Y | Y | Y | Y | Y |
| Brinkmann et al., 1988* | N | N | Y | N | Y | Y | Y | Y | N |
| Chaula & Tarimo, 2014* | Y | Y | Y | N | Y | Y | Y | Y | N |
| Garba et al., 2004* | Y | Y | Y | N | Y | Y | Y | Y | N |
| Hodges et al., 2012* | Y | Y | Y | N | Y | Y | Y | N | N |
| Hopkins et al., 2002 | Y | Y | Y | N | Y | Y | Y | N | Y |
| Janitschke et al., 1989* | N | N | Y | N | Y | Y | Y | N | N |
| Kaatano et al., 2015 | N | N | Y | N | Y | Y | Y | Y | N |
| Karanja et al., 2017* | Y | Y | Y | N | Y | Y | Y | Y | Y |
| Koukounari et al., 2007* | Y | Y | Y | N | Y | Y | Y | Y | N |
| Landoure et al., 2012* | Y | Y | Y | N | Y | Y | Y | Y | Y |
| Lin et al., 1997 | N | Y | Y | N | Y | Y | Y | Y | N |
| Massa et al., 2009* | Y | Y | Y | N | Y | Y | Y | Y | Y |
| Mduluza et al., 2001* | Y | N | N | N | Y | Y | Y | Y | N |
| Mwandawiro et al., 2019* | Y | Y | Y | N | Y | Y | Y | Y | Y |
| Mwinzi et al., 2012* | Y | Y | Y | N | Y | Y | Y | Y | N |
| N’Goran et al., 2001* | Y | Y | Y | N | Y | Y | Y | Y | N |
| Olsen et al., 2018* | Y | Y | Y | N | Y | Y | Y | Y | Y |
| Onkanga et al., 2016 | Y | Y | Y | Y | Y | Y | Y | Y | Y |
| Ouedraogo et al., 2016 | Y | Y | Y | N | Y | Y | Y | Y | Y |
| Pennance et al., 2016* | Y | Y | Y | N | Y | Y | Y | Y | Y |
| Phillips et al., 2017* | Y | Y | Y | N | Y | Y | Y | Y | N |
| Saathoff et al., 2004* | Y | Y | Y | N | Y | Y | Y | Y | N |
| Shehata et al., 2018* | Y | Y | Y | N | Y | Y | Y | Y | N |
| Stothard et al., 2009* | Y | Y | Y | N | Y | Y | Y | Y | N |
| Toure et al., 2008* | Y | Y | Y | N | Y | Y | Y | Y | Y |
| Wanjala et al., 2013* | Y | Y | Y | N | Y | Y | Y | Y | N |
| Zhang et al., 1998 | N | N | N | N | Y | Y | Y | Y | Y |
| Zhang et al., 2007* | Y | Y | Y | Y | Y | Y | Y | Y | Y |

* Study included in meta-analysis
