# Supplementary material for: Differential impact of mass and targeted praziquantel delivery on schistosomiasis control in school-aged children: A systematic review and meta-analysis
Source: PLoS Negl Trop Dis. 2019 Oct 11;13(10):e0007808. doi: 10.1371/journal.pntd.0007808 (PMC6808504; doi:10.1371/journal.pntd.0007808)
Supplement: S3 Table — (DOCX) [file pntd.0007808.s005.docx]

**S3 Table. Odds ratio of prevalence reduction for selected covariates, including treatment coverage, stratified by *Schistosoma* species (inverse variance weighted generalised linear model with robust error variance).**

| **Covariate** | **Odds ratio (95% CI)** | **p-value** | **R^2^** |
| --- | --- | --- | --- |
| ***Schistosoma mansoni*** | | | |
| Mass (n=7) v targeted (n=12) treatment | 0.50 (0.14-1.73) | 0.248 | 0.269 |
| Baseline prevalence (%) | 1.02 (0.89-1.06) | 0.307 |  |
| Number of treatment rounds | 0.73 (0.42-1.26) | 0.232 |  |
| Follow-up time (months) | 1.23 (1.00-1.51) | 0.053 |  |
| Treatment coverage |  |  |  |
| 75% or greater on average across treatment rounds | (ref) |  |  |
| Less than 75% on average across treatment rounds | 0.89 (0.16-5.11) | 0.890 |  |
| Not reported | 0.27 (0.08-0.95) | **0.043** |  |
| ***Schistosoma*** ***haematobium*** | | | |
| Mass (n=6) v targeted (n=13) treatment | 0.22 (0.12-2.58) | 0.209 | 0.3454 |
| Baseline prevalence (%) | 1.01 (0.95-1.07) | 0.799 |  |
| Number of treatment rounds | 0.64 (0.10-4.05) | 0.610 |  |
| Follow-up time (months) | 1.37 (0.58-3.25) | 0.438 |  |
| Treatment coverage |  |  |  |
| 75% or greater on average across treatment rounds | (ref) |  |  |
| Less than 75% on average across treatment rounds | 3.36 (0.01-901.68) | 0.647 |  |
| Not reported | 2.35 (0.34-16.42) | 0.359 |  |

**Bold** indicates a statistically significant result (p<0.05)
