# Supplementary material for: Differential impact of mass and targeted praziquantel delivery on schistosomiasis control in school-aged children: A systematic review and meta-analysis
Source: PLoS Negl Trop Dis. 2019 Oct 11;13(10):e0007808. doi: 10.1371/journal.pntd.0007808 (PMC6808504; doi:10.1371/journal.pntd.0007808)
Supplement: S4 Table — (DOCX) [file pntd.0007808.s006.docx]

**S4 Table. Odds ratio of prevalence reduction for selected covariates in sensitivity analysis, stratified by *Schistosoma* species (inverse variance weighted generalised linear model with robust error variance)**

|  | **Odds ratio (95% CI)** | **p-value** | **R^2^** |
| --- | --- | --- | --- |
| **Studies with >50% baseline prevalence excluded** | | | |
| ***Schistosoma mansoni*** | | | |
| Mass (n=5) v targeted (n=9) treatment | 0.06 (0.00–1.76) | 0.091 | 0.528 |
| Baseline prevalence (%) | 1.07 (0.94–1.21) | 0.275 |  |
| Number of treatment rounds | 1.08 (0.63–1.83) | 0.757 |  |
| Follow-up time (months) | 0.91 (0.56–1.47) | 0.672 |  |
| ***Schistosoma*** ***haematobium*** | | | |
| Mass (n=2) v targeted (n=7) treatment | 7.33 (0.86–62.13) | 0.061 | 0.977 |
| Baseline prevalence (%) | 1.00 (0.87–1.16) | 0.933 |  |
| Number of treatment rounds | 1.86 (1.12–3.09) | **0.027** |  |
| Follow-up time (months) | 12.84 (7.46–22.11) | **<0.001** |  |
| **Follow-up prevalence measured following one round of treatment (or closest)** | | | |
| ***Schistosoma mansoni*** | | | |
| Mass (n=7) v targeted (n=12) treatment | 0.12 (0.00–111.69) | 0.516 | 0.154 |
| Baseline prevalence (%) | 0.97 (0.87–1.10) | 0.700 |  |
| Number of treatment rounds | 2.76 (0.10–74.82) | 0.520 |  |
| Follow-up time (months) | 0.80 (0.43–1.51) | 0.468 |  |
| ***Schistosoma haematobium*** | | | |
| Mass (n=6) v targeted (n=13) treatment | 0.81 (0.08–8.52) | 0.847 | 0.070 |
| Baseline prevalence (%) | 1.00 (0.95–1.04) | 0.836 |  |
| Number of treatment rounds | 0.72 (0.38–1.38) | 0.296 |  |
| Follow-up time (months) | 1.03 (0.51–2.07) | 0.927 |  |

**Bold** indicates a statistically significant result (p<0.05)
