# Supplementary material for: Differential impact of mass and targeted praziquantel delivery on schistosomiasis control in school-aged children: A systematic review and meta-analysis
Source: PLoS Negl Trop Dis. 2019 Oct 11;13(10):e0007808. doi: 10.1371/journal.pntd.0007808 (PMC6808504; doi:10.1371/journal.pntd.0007808)
Supplement: S5 Table — (DOCX) [file pntd.0007808.s007.docx]

**S5 Table. Odds ratio of prevalence reduction for selected covariates, stratified by *Schistosoma* species (random effects weighted generalised linear model with robust error variance)**

|  | **Odds ratio (95% CI)** | **p-value** | **R^2^** |
| --- | --- | --- | --- |
| ***Schistosoma mansoni*** | | | |
| Mass (n=7) v targeted (n=12) treatment | 0.11 (0.01–2.48) | 0.152 | 0.257 |
| Baseline prevalence (%) | 1.03 (0.97–1.09) | 0.363 |  |
| Number of treatment rounds | 1.14 (0.56–2.34) | 0.700 |  |
| Follow-up time (months) | 1.00 (0.71–1.41) | 0.994 |  |
| ***Schistosoma haematobium*** | | | |
| Mass (n=6) v targeted (n=13) treatment | 0.06 (0.00–1.91) | 0.102 | 0.436 |
| Baseline prevalence (%) | 1.04 (0.98–1.10) | 0.144 |  |
| Number of treatment rounds | 0.93 (0.37–2.29) | 0.861 |  |
| Follow-up time (months) | 1.41 (0.65–3.05) | 0.360 |  |
