# Supplementary material for: Differential impact of mass and targeted praziquantel delivery on schistosomiasis control in school-aged children: A systematic review and meta-analysis
Source: PLoS Negl Trop Dis. 2019 Oct 11;13(10):e0007808. doi: 10.1371/journal.pntd.0007808 (PMC6808504; doi:10.1371/journal.pntd.0007808)
Supplement: S6 Table — (DOCX) [file pntd.0007808.s008.docx]

| **Delivery method** | **Number of doses** | **PReduc* (95% CI)** | **Cochran's Q** | ***p* value (Cochran’s Q)** | **Number of study datasets** |
| --- | --- | --- | --- | --- | --- |
| ***Schistosoma mansoni*** | | | | | |
| Mass | One dose | 0.01 (-0.76–0.66) | 27.63 | <0.001 | 4 |
|  | Multiple doses | 0.37 (0.18–0.52) | 36.55 | <0.001 | 3 |
| Targeted | One dose | 0.54 (0.42–0.63) | 84.34 | <0.001 | 7 |
|  | Multiple doses | 0.53 (0.26–0.70) | 235.49 | <0.001 | 5 |
| ***Schistosoma haematobium*** | | | | | |
| Mass | One dose | 0.64 (0.49–0.75) | 46.86 | <0.001 | 3 |
|  | Multiple doses | 0.14 (-0.26–0.42) | 111.41 | <0.001 | 3 |
| Targeted | One dose | 0.71 (0.53–0.83) | 216.34 | <0.001 | 7 |
|  | Multiple doses | 0.72 (0.47–0.85) | 574.68 | <0.001 | 6 |

**S6 Table. Meta-analysis results showing pooled prevalence reduction estimates (non-truncated) using random effects weights**

**PReduc* (prevalence reduction) = 1 – *PRatio*, where PRatio (prevalence ratio) = prevalence_follow-up_ /prevalence_baseline_
